# Response to Schoene et al.: Brain death determination during crisis requires ethical context and clinical perspective

**DOI:** 10.1186/s42466-025-00411-1

**Published:** 2025-09-25

**Authors:** Calixto Machado

**Affiliations:** Institute of Neurology and Neurosurgery, Havana, 10400 Cuba

## Abstract

This correspondence responds to Schoene et al.’s study on the impact of the COVID-19 pandemic on brain death (BD) detection in German hospitals. While their data-driven approach provides valuable insights, this response emphasizes the need to contextualize BD determination within the lived clinical and ethical realities of the pandemic. It argues that the reduction in BD assessments cannot solely be attributed to organizational lapses but must also account for ethical dilemmas, safety concerns, and triage pressures faced by healthcare providers. The author highlights the limitations of rigid BD protocols during public health emergencies and advocates for flexible, ethically guided practices. Drawing on international experience, including temporary adaptations in Cuba’s BD policies, the letter underscores the importance of physician support, context-sensitive decision-making, and humanistic engagement with families. Ultimately, it calls for a reexamination of how BD determinations are understood and implemented under crisis conditions, urging reforms that integrate medical standards with compassionate care and ethical reflection.

**To the editor,**

I read with interest the article by Schoene et al. [1], “Impact of the COVID-19pandemic on brain death detection in German hospitals.” The authors provide a valuable analysis of data from over 11,000 deceased patients with brain injury across three federal states, exploring how pandemic-related stressors affected the frequency of brain death (BD) determination and organ donation. While their use of regional COVID-19 incidence as a covariate in multilevel modeling adds analytical rigor, the findings raise broader questions about the clinical, ethical, and operational implications of BD protocols during public health emergencies [2–4]. As a physician who practiced throughout the pandemic and authored a response in Neurology highlighting the burdens faced by frontline clinicians, I wish to contribute a complementary perspective. The data offered by Schoene et al. must be situated within the lived experience of critical care medicine during the COVID-19 crisis—a period defined by resource exhaustion, moral injury, and triage decisions that often conflicted with the norms of end-of-life care [4, 5]. 

## The clinical perspective: complexity behind “missed” determinations

The authors frame a decline in BD determinations as a “detection deficit,” suggesting organizational failure or lapses in physician diligence. However, during peak pandemic phases, especially in the wild-type and alpha waves, many physicians faced an ethical and practical impossibility: evaluating BD in patients under strict infection control, under maximal ventilator dependency, or with profound hemodynamic instability [1]. 

For example, the apnea test—a cornerstone of BD diagnosis—was difficult or dangerous to perform in patients on ECMO or those with poor pulmonary reserves. Clinical uncertainty was compounded by the risk of aerosol-generating procedures. As such, decisions to delay or omit BD assessment were not oversights but informed clinical judgments prioritizing safety, palliative goals, or the futility of further diagnostics in deteriorating contexts [4, 5]. 

## The ethical burden: physician vulnerability and triage fatigue

The article briefly Acknowledges the mental health strain on healthcare workers but underemphasizes how this affected decision-making. In my experience and that of many colleagues, the early pandemic waves were marked by a pervasive sense of fear of transmitting SARS-CoV-2 to our families, of dying in isolation, and of abandoning professional obligations under impossible circumstances [4, 6]. 

Such psychological distress undermined the conditions for reflective clinical judgment. In this setting, pushing for formal BD determination—often in patients with no family present, no prior organ donation consent, and no realistic chance of recovery—could feel ethically fraught. The emphasis shifted from maximizing procurement to respecting the dignity of the dying, especially when palliative care resources were limited and family visitation was restricted [7, 8]. 

## The structural factor: absence of unified, flexible BD protocols

One of the key contributions of the Schoene et al. study is to quantify the fragility of BD determination processes under systemic stress. However, the root issue may not be COVID-19 incidence per se, but the rigidity of brain death protocols that lack adaptability in emergencies. In Germany, where donation after circulatory death (DCD) is not permitted, and where consent systems are opt-in, the entire organ procurement chain is highly dependent on strict BD confirmation. A more resilient system might have embraced context-sensitive criteria, perhaps with tiered determination strategies or conditional organ preservation measures until full evaluation could be safely completed.

In Cuba, for example, the national brain death protocol was temporarily adapted during the height of the pandemic to allow delayed confirmatory testing under ethical oversight, recognizing the limitations imposed by crisis conditions. This flexibility preserved the principle of BD while avoiding premature declarations or missed donations [9–11]. 

An Opportunity for Reframing: Beyond Data to Dialogue. Finally, I propose that what this study exposes is not merely a statistical deviation but a human reality: brain death, though medically defined, is contextually interpreted. The pandemic made this evident. Its stress tested not just ventilators and ICU beds, but our philosophical and cultural assumptions about life, death, and responsibility. We must resist interpreting BD frequency as a quality metric devoid of clinical narrative [12]. As the authors themselves concede, BD is a rare event; any shift, even with modest odds ratios, is statistically sensitive. But the ethical weight of aBD declaration—often made in the absence of family at the bedside, under time pressure and personal exhaustion—cannot be reduced to logistic regression. Instead, we should ask: What support do physicians need to make these declarations with confidence, clarity, and compassion? And what reforms in organ donation systems can account for human variability under stress [4, 5]? 

## Conclusion

Although the pandemic itself has subsided, it is imperative that we draw lasting lessons from the systemic limitations it revealed in medical care delivery. Emphasis must be placed on the diagnosis of brain death—not only to uphold the standards of neurological determination, but also to protect the well-being of the medical staff and to cultivate the tolerance and compassion required when supporting families during such profoundly delicate moments.

## Data Availability

Not applicable.
